# Tandospirone prevents stress-induced anxiety-like behavior and visceral hypersensitivity by suppressing theta oscillation enhancement *via* 5-HT_1A_ receptors in the anterior cingulate cortex in rats

**DOI:** 10.3389/fncel.2022.922750

**Published:** 2022-08-22

**Authors:** Ting-Ting Zhan, Zhi-Yu Dong, Li-Sha Yi, Yan Zhang, Hui-Hui Sun, Hai-Qin Zhang, Jun-Wen Wang, Ying Chen, Ying Huang, Shu-Chang Xu

**Affiliations:** ^1^Department of Gastroenterology, Tongji Institute of Digestive Diseases, Tongji Hospital, School of Medicine, Tongji University, Shanghai, China; ^2^Department of Gastroenterology, Zhangzhou Affiliated Hospital of Fujian Medical University, Zhangzhou, China; ^3^Key Laboratory of Spine and Spinal Cord Injury Repair and Regeneration (Ministry of Education), Department of Physiology and Pharmacology, Tongji Hospital, School of Medicine, Tongji University, Shanghai, China

**Keywords:** tandospirone, visceral hypersensitivity, theta oscillations, 5-HT_1A_ receptors, anterior cingulate cortex, stress, anxiety

## Abstract

Tandospirone, a third-generation of antianxiety agent with fewer side effects, has been widely used in the treatment of anxiety. Moreover, it is interesting that tandospirone has been found to relieve gastrointestinal symptoms in patients with refractory irritable bowel syndrome who also have psychological dysfunctions. However, the underlying mechanism remains unclear. In this study, using a visceral hypersensitivity rat model induced by chronic water avoidance stress to mimic the symptoms of irritable bowel syndrome, we found that tandospirone relieved anxiety-like behavior and visceral hypersensitivity induced by stress. Meanwhile, stressed rats had increased 5-HT concentration, less 5-HT_1A_ receptor expression, and enhanced theta oscillations in the anterior cingulate cortex (ACC). Furthermore, the power of the theta band in ACC is positively correlated with the level of visceral sensitivity. Activation of 5-HT_1A_ receptors by its agonist, 8-OH-DPAT, to compensate for their effect in ACC reduced the enhancement of theta oscillations in ACC slices in stressed rats, whereas 5-HT_1A_ receptor antagonist, WAY100135, facilitates theta oscillations in slices of normal rats. Tandospirone reduced the enhancement of theta band power in ACC *in vitro* and *in vivo*, thus alleviating anxiety-like behavior and visceral hypersensitivity through 5-HT_1A_ receptors in stressed rats. These results suggest a novel mechanism by which tandospirone activates 5-HT_1A_ receptors to relieve stress-induced anxiety and visceral hypersensitivity by suppressing theta oscillation enhancement in ACC.

## Introduction

Insights into gut-brain communication have revealed the interaction between the central and enteric nervous systems, linking emotional centers of the brain with peripheral intestinal function. Accumulating evidence suggests that psychological stress or emotional responses to stress have an impact on gastrointestinal function. Anxiety and depression are the most common psychiatric comorbidities of visceral pain in the prevalent gastrointestinal disorder, irritable bowel syndrome (IBS) (Whitehead et al., 2002). Clinical and animal studies have shown that anxiety or depression may induce visceral hypersensitivity, which is involved in the formation and aggravation of IBS symptoms (Wouters et al., 2012; Yi et al., 2014; Simrén et al., 2017). Some brain regions regulating visceral pain, such as the anterior cingulate cortex (ACC), insular cortex (IC), hippocampus, amygdala, and prefrontal cortex, are also involved in anxiety modulation (Elsenbruch et al., 2010; Mayer et al., 2019; Yi et al., 2019; Liu et al., 2020). This functional crosstalk has been proposed to underlie stress-induced anxiety and visceral hypersensitivity. Growing evidence established a link between stress-induced visceral hypersensitivity and abnormal activation in the limbic system, such as ACC, IC, and hippocampus (Elsenbruch et al., 2010; Wouters et al., 2012; Yi et al., 2014, 2019). An fMRI study showed that compared with healthy women, the female patients with IBS had a greater increase of neural activation in ACC and IC induced by non-painful rectal distensions under psychologic stress conditions (Elsenbruch et al., 2010). Besides, a PET study in maternal-separated rats after water avoidance stress (WAS) found activated clusters covering the hippocampus during colorectal distention (Wouters et al., 2012).

For IBS patients with psychological disorders, routine treatments such as dietary therapy, behavior modification, and gastrointestinal agents are ineffective. However, an additional application of psychopharmacological treatment is that it relieves gastrointestinal symptoms in these patients (Fukudo et al., 2021). For example, it has been found that anxiety remission alleviates gastrointestinal symptoms. A double-blind multicenter study reported that antispasmodic octatropine plus diazepam significantly reduced abdominal pain and discomfort of patients with IBS than placebo (Pace et al., 2010). Additionally, another randomized controlled trial in patients with IBS-diarrhea and moderate anxiety revealed that antispasmodic pinaverium plus tandospirone (TDS) alleviated abdominal pain, diarrhea, and anxiety in comparison to antispasmodic pinaverium plus placebo (Lan et al., 2014). TDS is a third-generation of antianxiety medicine that has fewer side effects, including sedation, cognitive impairment, and drug dependence, as compared to benzodiazepines (Suzuki et al., 1993; Evans et al., 1994; Nishitsuji et al., 2006). Therefore, it has been widely used in the treatment of anxiety caused by various neurosis and primary hypertension, functional dyspepsia, and other physical diseases accompanied by anxiety in China and Japan (Nishitsuji et al., 2004).

It has been well established that serotonin (5-HT) is an important neurotransmitter involved in multiple processes including the peristaltic reflex of the gastrointestinal tract and pathological states, such as pain disorders, anxiety, and depression (Primmer et al., 2010). As a 5-HT_1A_ receptor (5-HT_1A_R) partial agonist, TDS activated 5-HT_1A_Rs to exert its anxiolytic effect (Hamik et al., 1990). However, the potential mechanism of TDS relieving gastrointestinal symptoms remains to be elucidated. In this study, using behavioral tests, electrophysiological, and molecular biology techniques, we found that TDS alleviated stress-induced visceral hypersensitivity *via* ACC-5-HT_1A_R-theta oscillation-dependent inhibition of central sensitization.

## Materials and methods

### Animals

Adult male Wistar rats (200–250 g) were acquired from Shanghai Sippr-BK Laboratory Animal Co., Ltd. All rats were housed in groups of four in cages with a 12-h light/dark cycle and free access to food and drink. After 7 days of acclimation, rats were subjected to subsequent experiments. Six to eight rats were randomly assigned to each group. All rats were enrolled except for the rats that died before the end of the experiment. All experimental procedures were performed according to the procedures approved by the Animal Care and Use Committee of Tongji University.

### Visceral hypersensitivity rat model

Chronic WAS (Stressed) was used to induce visceral hypersensitivity in rat models as previously described (Yi et al., 2019). In brief, an 8 cm length × 8 cm width × 10 cm height vertical platform was placed in the center of a 25 cm length × 25 cm width × 45 cm height water tank. The surface of the water was 1 cm below the top of the platform. The WAS rats were placed on the platform for 1 h every day (between 9 a.m. and 12 a.m.) for 10 consecutive days. The normal control (Unstressed) rats were treated without any intervention.

### Assessment of visceromotor response

By recording an abdominal muscle electromyogram (EMG), the visceromotor response (VMR) to colorectal distension (CRD) was monitored to measure the visceral sensitivity of rats (Yi et al., 2019). Briefly, a pair of Teflon-coated stainless wires were surgically implanted into the left external abdominal oblique muscles of rats. After surgery, the rats were housed separately 3 days before EMG was recorded. On the test day, a flexible latex balloon (4 cm long) tied around a urethral catheter (3 mm diameter) was inserted into descending colon with the distal tip 1 cm from the anal verge under mild anesthesia. The 60 mmHg CRD was performed by rapidly injecting gas into the colonic balloon within 1 s and holding the distention for 20 s. Three cycles of CRD (20-s duration; 5-min inter-stimulus interval) were applied to each rat. The EMG signal was recorded using 4-channel Brownlee Precision Model 440 Amplifier (Brownlee Precision, United States) and Axon Digidata 1322A data acquisition system (Molecular Devices, United States). The EMG signal was amplified (×5,000), band-pass filtered at (50–500 Hz), and sampled at 1 kHz. The EMG signal was quantified by calculating the difference between the area under the curve (AUC) of baseline and the CRD period using Clamp Fit 10.7 (Molecular Devices, United States).

### Behavioral experiments

#### Open field test

To evaluate the anxiety level of rats, the open field test (OFT) was performed as previously described (Yi et al., 2019). In brief, rats were subjected to OFT in a bright room (between 9 a.m. and 3 p.m.) and were individually placed in the center of an opaque box with a black bottom and white walls (40 cm length × 40 cm width × 40 cm height). Before the experiment, rats were brought into the testing room in their home cages and allowed to acclimate to the testing environment for 30 min. The movement of each rat in the first 5 min was recorded by a camera above the box. Behavioral parameters including total traveled distance, frequency, and duration of entries into the center zone, and the number of rearing were analyzed using the ANY-maze system (Stoelting Co., United States). The open field was swabbed with 70% ethanol after each rat was tested.

#### Elevated plus maze

The elevated plus maze (EPM) test was performed to assess the anxiety level of rats as previously described (Yi et al., 2019). The EPM was made up of two relatively open arms (50 cm length × 10 cm width), two relatively closed arms (50 cm length × 10 cm width × 40 cm height), a central area (10 cm × 10 cm), and a metal frame that raised the apparatus 60 cm off the ground. Briefly, rats were individually placed into the central area facing a closed arm. The movement of each rat in the first 5 min was recorded by a camera above the EPM. Behavioral parameters including the percentage of frequency of entries into the open arms and percentage of duration in the open arms were analyzed using the ANY–maze system (Stoelting Co., United States). The EPM was swabbed with 70% ethanol after each rat was tested.

#### Enzyme-linked immunosorbent assay

Anterior cingulate cortex tissue was homogenized mechanically on ice with lysis buffer and then centrifuged at 1,000 *g* for 20 min at 4^°^C. The supernatant was collected for the Enzyme-Linked Immunosorbent Assay (ELISA). The ELISA kit was used to quantitatively analyze the 5-HT concentration following the manufacturer’s instructions provided in the kit (5-Hydroxytryptamine, catalog no. CEA808Ge, Cloud-clone).

#### Real-time quantitative PCR

The total RNA of the ACC tissues was extracted using the Trizol Reagent (catalog no. 15596026, Invitrogen) according to the manufacturer’s instructions. Then, the cDNA was synthesized using the PrimeScript™ RT Master Mix Kit (catalog no. RR036A, Takara). The expression of target genes was analyzed using the TB Green^®^ Premix Ex Taq™ Kit (catalog no. RR420A, Takara) by the QuantStudio 7 Flex Real-Time PCR System (Applied Biosystems, United States). The mRNA expression was quantified using the ΔΔCt method and normalized against the GAPDH expression level. Primer sequences were as follows:

GAPDH (F: 5′-ACGGCAAGTTCAACGGCACAG-3′; R:5′-CGACATACTCAGCACCAGCATCAC-3′).
Serotonin 1A receptors (5-HT_1A_Rs) (F:5′-AGGACCA CGGCTACACCATCTAC-3′; R:5′-CTGACAGTCTTGCG GATTCGGAAG-3′).
Serotonin 1B receptors (5-HT_1*B*_Rs) (F:5′-CACTG ATGCGGTGGACTATTCTGC-3′; R:5′-CGTGGAGTAG ACCGTGTAGAGGAC-3′).
Serotonin 1F receptors (5-HT_1*F*_Rs) (F:5′-TGGCTGA GTGTTGACATCATCTGC-3′; R:5′-CTGCGTCTGTGA TTGCTCGGTAC-3′).
Serotonin 2A receptors (5-HT_2A_Rs) (F:5′-CCGCTATGT CGCCATCCAGAAC-3′; R:5′-ACAGATATGGTCCACAC GGCAATG-3′).
Serotonin 2B receptors (5-HT_2*B*_Rs) (F:5′-CAACGCCTAA CACGGTGGACTG-3′; R:5′-CCTTGTGAGAGCCATCC AGCATC-3′).
Serotonin 2C receptors (5-HT_2*C*_Rs) (F:5′-GGTCCTTCGT GGCATTCTTCATCC-3′; R:5′-TTAGCCAGTTCCTCCT CGGTGTG-3′).
Serotonin 3 receptors (5-HT_3_Rs) (F:5′-CTGTCCTCCA TCCGCCACTCC-3′; R:5′-CAGCAGCCTGTCCAGCAC ATATC-3′).
Serotonin 4 receptors (5-HT_4_Rs) (F:5′-AAGGCTGGA ACAACATCGGCATAG-3′; R:5′-CACAGAGCAGGTGA TGGCATAGG-3′).
Serotonin 5A receptors (5-HT_5A_Rs) (F:5′-TACGCACC TTCCACCGAGTACC-3′; R:5′-TACCAGGCTCAGAGG CATAACCAG-3′).
Serotonin 6 receptors (5-HT_6_Rs) (F:5′-TGACAGCAGC CGCCAATTCG-3′; R:5′-CACCATCAAGTCCGACGTG AAGAG-3′).
Serotonin 7 receptors (5-HT_7_Rs) (F:5′-TTCTGTCGGT CTGGCTGCTCTC-3′; R:5′-ACCGCAGTGGAGTAGAT CGTGTAG-3′).

#### Western blot

Total protein of the ACC tissues was extracted using RIPA buffer supplemented with a protease inhibitor cocktail. Then, the concentration of protein was determined using the BCA Protein Assay Kit (catalog no. P0010S, Beyotime). Subsequently, 30 μg of protein was loaded per lane. After gel electrophoresis, membrane transfer, and blocking, the membranes were immunoblotted with primary antibodies against 5-HT_1A_Rs (1:1,000, catalog no. ab85615, Abcam), 5-HT_2A_Rs (1:100, catalog no. sc-166775, Santa Cruz), 5-HT_7_Rs (1:1,000, catalog no. ab128892, Abcam), and GAPDH (1:10,000, catalog no. G9295, Sigma-Aldrich) at 4^°^C overnight. After washing, the membranes were incubated with horseradish peroxidase-labeled goat anti-rabbit IgG antibody (1:2,000, catalog no. ARG65351, Arigo) or horseradish peroxidase-labeled goat anti-mouse IgG antibody (1:2,000, catalog no. ARG65350, Arigo) at room temperature for 2 h. Membranes were revealed using enhanced chemiluminescence reagents (ECL, catalog no. WBKLS0010, Millipore) by a chemiluminescence imaging system (GE Healthcare, United States). The protein expression levels were quantified based on the intensities of the bands with the Image J software and normalized against the GAPDH expression level. Additionally, the membranes reacted with 5-HT receptor antibodies, then stripped, and re-probed with GAPDH antibody in western blot analysis.

#### Recording of local field potentials in anterior cingulate cortex *in vitro*

Coronal slices (500 μm thick, Bregma ∼3.2-1.7) containing the ACC were prepared as previously described (Steullet et al., 2014). Briefly, rats were decapitated under diethyl ether anesthesia. Then brains were rapidly removed and immersed in cold oxygenated cutting solution (in mM: 251.8 sucrose, 2.5 KCl, 4.0 MgCl_2_, 0.5 CaCl_2_, 1.2 NaH_2_PO_4_, 23.8 NaHCO_3_, and 11.0 glucose; pH 7.4). The ACC slices were prepared with a Leica VT1000S vibratome (Leica Instruments, China) in a cold oxygenated cutting solution. Then, slices were incubated in oxygenated artificial cerebrospinal fluid (ACSF) (in mM: 123.9 NaCl, 3.8 KCl, 2.0 MgCl_2_, 2.0 CaCl_2_, 1.3 NaH_2_PO_4_, 23.8 NaHCO_3_, 11.0 glucose; pH 7.4; 25–27°C) and transferred to a submerged recording chamber double surfaces perfused at a rate of 6.0–7.0 ml/min with oxygenated ACSF (in mM: 123.9 NaCl, 5.0 KCl, 1.5 MgCl_2_, 2.0 CaCl_2_, 1.3 NaH_2_PO_4_, 23.8 NaHCO_3_, 11.0 glucose; pH 7.4; 28–29°C). Electrophysiological recordings were made using the MultiClamp 700B amplifier (Molecular Devices, United States) and Axon Digidata 1550B data acquisition system (Molecular Devices, United States) at least 2 h after slicing. The local field potentials (LFPs) signals were recorded with ACSF-filled glass electrodes (3–4 MΩ) at a sample rate of 10 kHz in the layers II/III principal neurons of ACC. Interference of 50 Hz was filtered by HumBug (Quest Scientific, Canada). The theta oscillations in ACC were generated with kainic acid (KA, 0.8 μM, catalog no. K0250, Sigma-Aldrich) and carbachol (CCh, 50 μM, catalog no. C4382, Sigma-Aldrich) (Steullet et al., 2014). The baseline LFPs were recorded for 1 min prior to drug application. The following signals were recorded for at least 10 min during drug application. 8-hydroxy-2-(di-n-propylamino) tetralin (8-OH-DPAT, 20 μM, Sigma-Aldrich), N-tert-Butyl-3-(4-(2-methoxyphenyl)-piperazin-1-yl)-2-phenylpropanamide dihydrochloride (WAY100135, 10 μM, Sigma-Aldrich), or TDS (20 μM, Sumitomo Dainippon Pharma Co.) were applied in the perfusion according to previous reports (Kazmierska and Konopacki, 2015). The fast Fourier transformation was performed to calculate the power spectra and integral power of theta oscillations (4–10 Hz) for 60-s-recordings by Clamp Fit 10.7. Only the recordings with a stable baseline were included in the data analysis.

#### Recording of local field potentials in anterior cingulate cortex *in vivo*

Rats were anesthetized with pentobarbital (50 mg/kg, i.p.) and placed in a stereotaxic frame. The recording electrodes were implanted into the ACC (AP + 2.7 mm, *L* ± 0.6 mm, *V* – 2.5 mm). The silver grounding wires from the recording electrodes were wrapped around the mounting screws. A mixture of paraffin oil and bone wax was packed around the electrode penetration zone. Then electrodes were secured to the skull with screws and dental cement. After surgery, rats were housed separately for a 3-day recovery. TDS was dissolved in phosphate-buffered saline (PBS) and prepared at a concentration of 10 mg/ml. The rats were intraperitoneally injected with TDS or vehicle in a volume of 1 ml/kg, at a time of 30 min before the WAS stimulation every day (Nishitsuji et al., 2006; Murata et al., 2015). When WAS halted on day 11, TDS administration was stopped accordingly. All rats were allowed to adapt to the recording environment and were handled for 5 min daily for 3 days before ACC LFPs and EMG recordings. The LFPs were recorded using the 4-channel Brownlee Precision Model 440 Amplifier and Axon Digidata 1322A digitizer. LFPs were amplified (×10,000), band-pass filtered (0.05–100 Hz), and sampled at 1 kHz. Recordings were obtained during quiet and awake states. Each rat was recorded in the 20 s before CRD, 20 s during CRD, and 20 s after CRD during quiet and awake states. The fast Fourier transformation was performed to calculate the power spectra and integral power of theta oscillations (4–10 Hz) by Clamp Fit 10.7. The time–frequency diagrams were performed by short-time Fourier transformation using custom-written Matlab (MathWorks, United States). After recordings, histological staining was conducted to confirm the location of the recordings. The data obtained from the rats with recording sites outside the ACC region were excluded for final analysis.

#### Microinjection in anterior cingulate cortex

After the rats were anesthetized, a stainless-steel guide cannula (3.5 mm) was implanted into the ACC region (AP + 2.7 mm, *L* ± 0.6 mm, *V* – 2.5 mm) just like the procedures of recording electrodes implantation *in vivo*. A stylet (3.5 mm) was inserted in the guide cannula to prevent clogging. After the surgery, rats were housed separately for a 3-day recovery. Microinjections were performed using an injection cannula extending 0.5 mm beyond the tip of the guide cannula under mild anesthesia. The injection cannula was attached to a microliter syringe (Hamilton, United States) through a plastic tube. N-{2-[4-(2-methoxyphenyl)-1-piperazinyl]ethyl}-N-2-pyridinylcyclohexane carboxamide (WAY100635, Sigma-Aldrich, 30 nmol/0.2 μl PBS) or PBS (0.2 μl) was infused over a 60-s period at a rate of 0.2 μl/min with the help of syringe pump (Harvard Apparatus, United States) (Sartim et al., 2016). After 15 min, the rats were intraperitoneally injected with TDS, and then WAS stimulation was started 30 min later (Nishitsuji et al., 2006; Murata et al., 2015). Histological staining was conducted to confirm the location of the microinjection. The data obtained from the rats with cannulation sites outside the ACC region were excluded for final analysis.

### Histological analysis

After completion of ACC LFPs recording *in vivo*, rats were anesthetized with sodium pentobarbital (50 mg/kg, i.p.) and then a DC current (100 μA, 15 s) was given to the lesion in the brain to mark the recording sites. Similarly, at the end of experiments of microinjection, rats were anesthetized and perfused intracardially with PBS, followed by a fixative containing 4% paraformaldehyde. The brain sections were stained with Nissls staining to assess the recording sites according to the atlas of the rat brain. The data obtained from the rats with cannulation or recording sites outside the ACC region were excluded for final analysis.

### Statistical analysis

Data were tested for normality and expressed as mean ± SE. Comparison between the two groups was performed by the two-tailed unpaired Student’s *t*-test. Multiple comparisons were performed by one-way ANOVA followed by the Bonferroni test. SPSS 17.0 was used for statistical analysis.

## Results

### Tandospirone ameliorated visceral hypersensitivity and anxiety-like behavior in stressed rats

**FIGURE 1:**
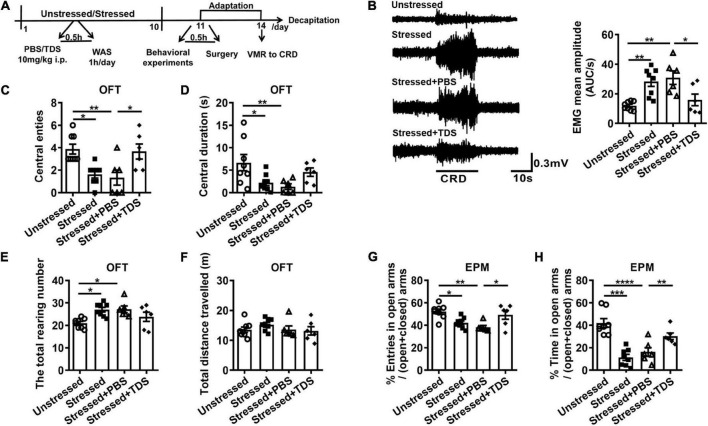
Tandospirone (TDS) ameliorated visceral hypersensitivity and anxiety-like behavior in stressed rats. **(A)** Experimental design. **(B)** The representative EMG of visceromotor response to 60 mmHg CRD (left) and the mean amplitude of EMG (right). Analysis of the number of central entries **(C)**, central duration **(D)**, the total raring number **(E)**, the total distance travelled **(F)** in the open field test (OFT). Analysis of the percentage of a number of open arms entries **(G)** and the percentage of time spent into open arms **(H)** in the elevated plus maze (EPM) test. Unstressed: *n* = 8, Stressed: *n* = 8, Stressed + PBS: *n* = 6, Stressed + TDS: *n* = 6, *p < 0.05, **p < 0.01, ***p < 0.001, ****p < 0.0001. Data are presented as means ± SEM.

To determine the effect of TDS on stress-induced visceral hypersensitivity, we examined VMR and anxiety-like behavior in stressed rats with or without TDS intraperitoneal injection (Figure 1A). We found the VMR amplitude of stressed and stressed + PBS rats increased compared with that of unstressed rats. TDS application reduced the enhancement of VMR amplitude in stressed rats (Unstressed: 11.9 ± 1.0 AUC/s, Stressed: 28.3 ± 3.3 AUC/s, Stressed + PBS: 30.9 ± 4.6 AUC/s, Stressed + TDS: 15.9 ± 3.9 AUC/s, Stressed vs. Unstressed: *p* < 0.01, Stressed + PBS vs. Unstressed: *p* < 0.01, and Stressed + TDS vs. Stressed + PBS: *p* < 0.05; Figure 1B). Animals avoiding the central areas in OFT and open arms in EPM were considered more anxious. In the OFT, stressed and stressed + PBS rats showed fewer number of central entries (Unstressed: 3.9 ± 0.4, Stressed: 1.6 ± 0.3, Stressed + PBS: 1.3 ± 0.7, Stressed vs. Unstressed: *p* < 0.05, and Stressed + PBS vs. Unstressed: *p* < 0.01; Figure 1C), shorter central duration (Unstressed: 6.7 ± 1.8 s, Stressed: 2.2 ± 0.7 s, Stressed + PBS: 1.3 ± 0.6 s, Stressed vs. Unstressed: *p* < 0.05, and Stressed + PBS vs. Unstressed: *p* < 0.01; Figure 1D), and more raring number (Unstressed: 21.0 ± 0.8, Stressed: 27.0 ± 1.0, Stressed + PBS: 27.2 ± 1.5, Stressed vs. Unstressed: *p* < 0.05, and Stressed + PBS vs. Unstressed: *p* < 0.05; Figure 1E) than that of unstressed rats. In the EPM, stressed rats showed less percentage of number of open arms entries (Unstressed: 52.3 ± 2.2%, Stressed: 42.2 ± 1.7%, Stressed + PBS: 38.3 ± 1.6%, Stressed vs. Unstressed: *p* < 0.05, and Stressed + PBS vs. Unstressed: *p* < 0.01; Figure 1G) and spent less percentage of time in open arms (Unstressed: 42.0 ± 3.9%, Stressed: 11.4 ± 2.7%, Stressed + PBS: 16.2 ± 3.9%, Stressed vs. Unstressed: *p* < 0.001, and Stressed + PBS vs. Unstressed: *p* < 0.0001; Figure 1H) when compared with unstressed rats, suggesting stressed rats were more anxious. TDS application relieved the reduction of central entries (Stressed + TDS: 3.7 ± 0.7 and Stressed + PBS: 1.3 ± 0.7, *p* < 0.05; Figure 1C) in the OFT, the percentage of number of open arms entries (Stressed + TDS: 49.3 ± 3.9% and Stressed + PBS: 38.3 ± 1.6%, *p* < 0.05; Figure 1G) and the percentage of time spent in open arms (Stressed + TDS: 30.2 ± 2.9%, Stressed + PBS: 16.2 ± 3.9%, *p* < 0.01; Figure 1H) in the EPM of stressed rats. These results suggest that TDS ameliorates visceral hypersensitivity and anxiety-like behavior induced by water avoidance stress.

### Stress increased 5-HT concentration, reduced 5-HT_1A_R expression, and enhanced theta band power in anterior cingulate cortex

**FIGURE 2:**
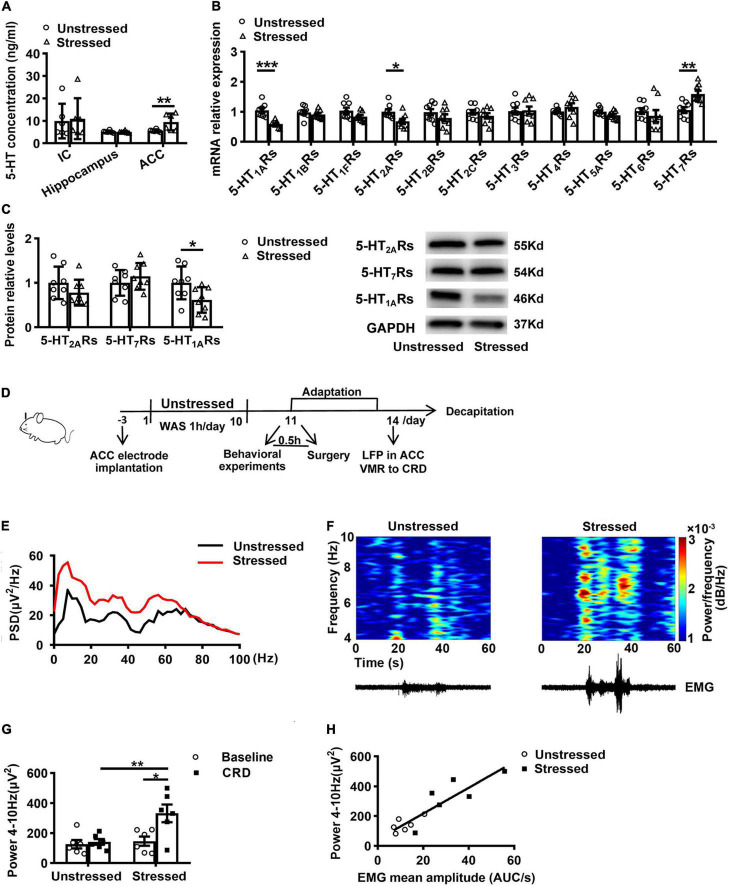
Stress increased 5-HT concentration, reduced 5-HT_1A_Rs expression, and enhanced theta band (4–10 Hz) power in ACC. **(A)** 5-HT concentration in the insular cortex (IC), hippocampus, and anterior cingulate cortex (ACC) (*n* = 6 for Unstressed and Stressed respectively). **(B)** mRNA expression of serotonin receptor gene in ACC (*n* = 8 for Unstressed and Stressed, respectively). **(C)** Western blot analysis of the serotonin receptor protein in ACC (*n* = 8 for Unstressed and Stressed respectively). Stress enhanced theta band power in ACC *in vivo* (*n* = 6 for Unstressed and Stressed, respectively). **(D)** Experimental design. **(E)** Mean power spectral density (PSD) plots during 20s-CRD treatment in ACC of unstressed (black line) and stressed (red line) rats. **(F)** Time–frequency diagrams of the power of theta oscillations in ACC and corresponding EMG. **(G)** The integral power of theta oscillations during pre-CRD (Baseline) and 20s-CRD. **(H)** Correlation between theta oscillation power in ACC during 20s-CRD and the EMG of visceromotor response, based on combined data points obtained in the unstressed (circle) and stressed (square) rats, *R*^2^ = 0.8019, *p* < 0.0001. *p < 0.05, **p < 0.01, ***p < 0.001. Data are presented as means ± SEM.

As a 5-HT system modulator, TDS activates 5-HT_1A_Rs partially and relieves anxiety-like behavior (Hamik et al., 1990; Nishitsuji et al., 2004). To investigate whether the 5-HT system involves in stress-induced visceral hypersensitivity and which brain regions mediate this effect, we first examined 5-HT concentration in ACC, IC, and hippocampus, which are important regions in regulating visceral sensitivity and chronic stress (Jovanovic et al., 2011; Wouters et al., 2012; Yi et al., 2014). ELISA results revealed that 5-HT concentration in IC and hippocampus were not different between unstressed and stressed rats, whereas the 5-HT concentration in ACC of stressed rats was increased (Stressed: 9.6 ± 1.5 ng/ml, Unstressed: 5.6 ± 0.2 ng/ml, *p* < 0.05; Figure 2A). Moreover, we compared the levels of 5-HT receptor mRNA and protein in ACC between unstressed and stressed rats. qPCR results showed that the mRNA expression of 5-HT_1A_Rs and 5-HT_2A_Rs was reduced, while the 5-HT_7_Rs mRNA was upregulated in ACC of stressed rats (Figure 2B). Furthermore, the reduction of 5-HT_1A_Rs proteins was confirmed by western blot analysis, but the change of 5-HT_2A_Rs or 5-HT_7_Rs protein was not detected (Figure 2C). These results suggest that stress facilitates 5-HT release but reduces 5-HT_1A_Rs expression in ACC.

It has been found in fMRI studies that patients with IBS had enhanced activity in ACC (Mertz et al., 2000). LFP recording is able to be used to monitor neural activity in humans with more detailed information than fMRI (Einevoll et al., 2013). To investigate the further mechanisms underlying the change of ACC activity in stressed rats, we recorded LFPs in ACC and VMR to CRD simultaneously in unstressed and stressed rats *in vivo* (Figure 2D). Power spectral analysis showed the powers of LFPs at theta band (4–10 Hz) in ACC during CRD were enhanced (Figure 2E). Compared with unstressed rats, the integral power of theta oscillations during 20 s-CRD in stressed rats was increased (Stressed: 331.8 ± 59.0 μV^2^, Unstressed: 141.5 ± 19.4 μV^2^, *p* < 0.01; Figure 2G). Moreover, only the stressed rats showed notably enhanced theta band power during CRD than baseline (Baseline: 146.7 ± 30.2 μV^2^, CRD: 331.8 ± 59.0 μV^2^, *p* < 0.05; Figure 2G). The time–frequency diagrams of the power of theta oscillations in ACC and the corresponding VMR to CRD are shown in Figure 2F. The enhanced theta band power in ACC is temporally coupled with the enhancement of VMR induced by 60 mmHg CRD. Further linear regression analysis showed that the theta band power in ACC during 20 s-CRD was positively correlated with the VMR to CRD (*R*^2^ = 0.8019, *p* < 0.0001; Figure 2H). These results suggest the connection of enhancement of theta band LFPs in ACC with visceral hypersensitivity in stressed rats.

### 5-HT_1A_Rs regulated theta oscillations in anterior cingulate cortex of stressed rats

**FIGURE 3:**
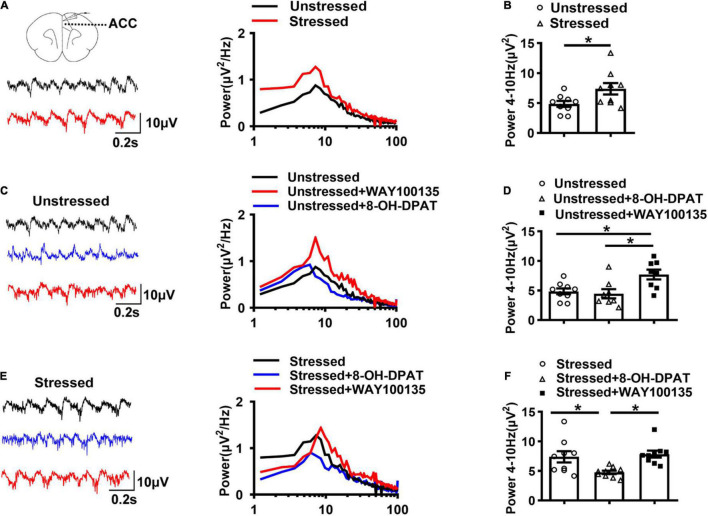
5-HT_1A_Rs regulated theta oscillations (4–10 Hz) in ACC of stressed rats. **(A)** Sample traces (left) and power spectra (right) of oscillations induced by 50 μM carbachol (CCh) and 0.8 μM kainic acid (KA) in ACC slices of unstressed (black line) and stressed (red line) rats. **(B)** The integral power of theta oscillations in ACC (Unstressed: *n* = 9 from seven rats, Stressed: *n* = 9 from five rats). **(C)** Sample traces (left) and power spectra (right) of oscillations in ACC slices of unstressed rats in the presence of 8-OH-DPAT (20 μM) or WAY100135 (10 μM). **(D)** Summary of the data showing the effect of 8-OH-DPAT and WAY100135 on theta oscillations in ACC of unstressed slices (Unstressed: *n* = 9 from six rats, Unstressed + 8-OH-DPAT: *n* = 8 from six rats, Unstressed + WAY100135: *n* = 8 from five rats). **(E)** Sample traces (left) and power spectra (right) of oscillations in ACC slices of stressed rats in the presence of 8-OH-DPAT (20 μM) or WAY100135 (10 μM). **(F)** Summary of the data showing the effect of 8-OH-DPAT and WAY100135 on theta oscillations in ACC of stressed slices (Stressed: *n* = 9 from five rats; Stressed + 8-OH-DPAT: *n* = 9 from six rats; Stressed + WAY100135: *n* = 9 from six rats). *p < 0.05. Data are presented as means ± SEM.

To further explore the underlying mechanisms of TDS relieving visceral hypersensitivity without the influence of breathing and heartbeat, we also examined the changes of LFPs in brain slices *in vitro*. Theta oscillations were induced by perfusion of 50 μM CCh and 0.8 μM KA in ACC slices. The results showed that the theta band power in ACC of stressed rats was increased than that of unstressed rats (Stressed: 7.4 ± 1.0 μV^2^, Unstressed: 4.9 ± 0.5 μV^2^, *p* < 0.05; Figures 3A,B), which further confirmed the enhancement of theta band LFPs in ACC of stressed rats *in vivo*.

Given the higher concentration of 5-HT and less expression of 5-HT_1A_Rs and enhanced theta band LFPs in ACC of stressed rats that we found, we further compared the CCh-KA-induced theta oscillations in the presence of 5-HT_1A_Rs agonists or antagonists in ACC slices to explore the relation between 5-HT_1A_Rs and theta oscillations. The results showed that 5-HT_1A_Rs antagonist, WAY100135 (10 μM), increased the theta band power of ACC in slices of unstressed rats, which mimicked the change in stressed rats, while 5-HT_1A_Rs agonist, 8-OH-DPAT (20 μM), did not change the theta band power of ACC in unstressed rats (Unstressed: 4.9 ± 0.5 μV^2^, Unstressed + 8-OH-DPAT: 4.5 ± 0.8 μV^2^, Unstressed + WAY100135: 7.7 ± 0.8 μV^2^, Unstressed vs. Unstressed + WAY100135: *p* < 0.05, Unstressed + 8-OH-DPAT vs. Unstressed + WAY100135: *p* < 0.05; Figures 3C,D). In stressed rats, 8-OH-DPAT decreased the theta band power of ACC, while WAY100135 did not show remarkable effect on theta band power of ACC (Stressed: 7.4 ± 1.0 μV^2^, Stressed + 8-OH-DPAT: 4.8 ± 0.3 μV^2^, Stressed + WAY100135: 7.8 ± 0.6 μV^2^, Stressed vs. Stressed + 8-OH-DPAT: *p* < 0.05, Stressed + 8-OH-DPAT vs. Stressed + WAY100135: *p* < 0.05; Figures 3E,F). These results suggest that enhanced theta oscillations in ACC are associated with inhibition of 5-HT_1A_Rs and vice versa.

### Tandospirone reduced the enhancement of theta band power in anterior cingulate cortex of stressed rats

**FIGURE 4:**
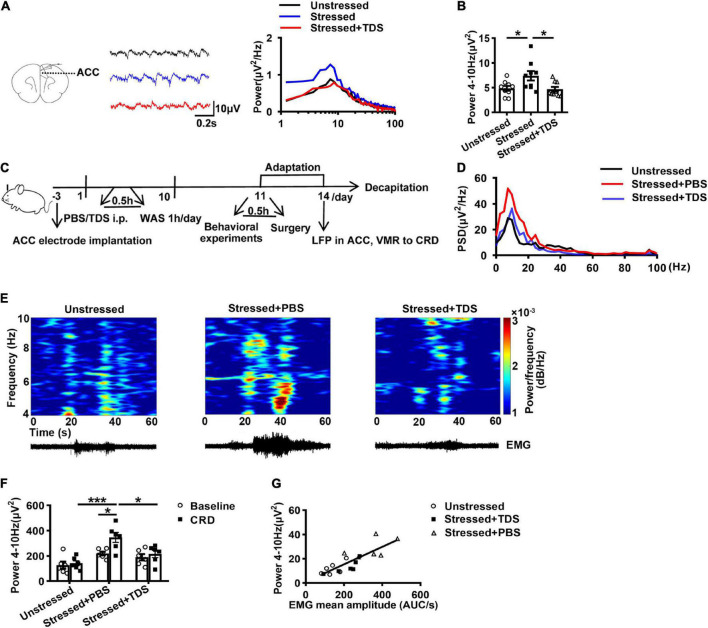
TDS reduced the enhancement of theta band (4–10 Hz) power in ACC of stressed rats. **(A)** Sample traces (left) and power spectra (right) of oscillations in ACC slices in the absence or presence of TDS. **(B)** Summary of the data showing the effect of TDS on theta oscillations in ACC of stressed rats (Unstressed: *n* = 9 from six rats, Stressed: *n* = 9 from five rats, Stressed + TDS: *n* = 9 from five rats). TDS reduced the enhancement of theta band power in ACC of stressed rats *in vivo* (Unstressed, Stressed + PBS, and Stressed + TDS: *n* = 6, respectively). **(C)** Experimental design. **(D)** Mean power spectral density (PSD) plots during 20s-CRD treatment in ACC of unstressed (black line), stressed + PBS (red line), and stressed + TDS (blue line) rats. **(E)** Time-frequency diagrams of the power of theta oscillations in ACC and corresponding EMG. **(F)** The integral power of theta oscillations during pre-CRD (Baseline) and 20s-CRD. **(G)** Correlation between theta oscillation power in ACC during 20s-CRD and the EMG of visceromotor response, based on combined data points obtained in the unstressed (circle), stressed + PBS (triangle) and stressed + TDS (square) rats, *R*^2^ = 0.6908, *p* < 0.0001. *p < 0.05, **p < 0.01, ***p < 0.001. Data are presented as means ± SEM.

Because of intolerable gastrointestinal side effects, 8-OH-DPAT is not applied to humans (Savitz et al., 2009). TDS is a 5-HT_1A_Rs partial agonist commonly used in clinical treatment. Due to the above results, we further examined the effect of TDS on theta oscillations in ACC of WAS rats. First, we applied TDS on WAS slices, and the results showed that TDS reduced the enhancement of theta band power in ACC of stressed rats (Stressed + TDS: 4.7 ± 0.5 μV^2^, Stressed: 7.4 ± 1.0 μV^2^, Unstressed: 4.9 ± 0.5 μV^2^, Stressed + TDS vs. Stressed: *p* < 0.05, and Stressed vs. Unstressed: *p* < 0.05; Figures 4A,B).

Additionally, to clarify the effect of TDS on theta oscillations in ACC and visceral sensitivity *in vivo*, we further monitored the LFPs of ACC and VMR to CRD simultaneously in stressed rats after intraperitoneal injection of TDS. The corresponding control group received an intraperitoneal injection of PBS under the same conditions (Figure 4C). Consistently, in the control group, the theta band power in ACC of stressed rats was increased compared with that of unstressed rats. TDS application reduced the enhancement of theta oscillations in stressed rats (Stressed + TDS: 215.2 ± 28.9 μV^2^, Stressed + PBS: 346.0 ± 39.3 μV^2^, Unstressed: 141.5 ± 19.4 μV^2^, Stressed + PBS vs. Unstressed: *p* < 0.001, and Stressed + TDS vs. Stressed + PBS: *p* < 0.05; Figures 4D–F). Only the stressed + PBS group showed notably enhanced theta band power during CRD than baseline (Baseline: 218.7 ± 14.7 μV^2^ vs. CRD: 346.0 ± 39.3 μV^2^, *p* < 0.05; Figure 4F). Moreover, the linear regression analysis showed that the theta band power in ACC during 20 s-CRD was positively correlated with the VMR to CRD (*R*^2^ = 0.6908, *p* < 0.0001; Figure 4G). The reduction of theta band power in ACC during 60 mmHg CRD in stressed + TDS rats occurred in synchrony with the decline of VMR. These *in vivo* results suggest that TDS reducing the enhancement of theta oscillations in ACC may contribute to its amelioration of visceral hypersensitivity in stressed rats.

### The effects of tandospirone in stressed rats were mediated by 5-HT_1A_Rs

**FIGURE 5:**
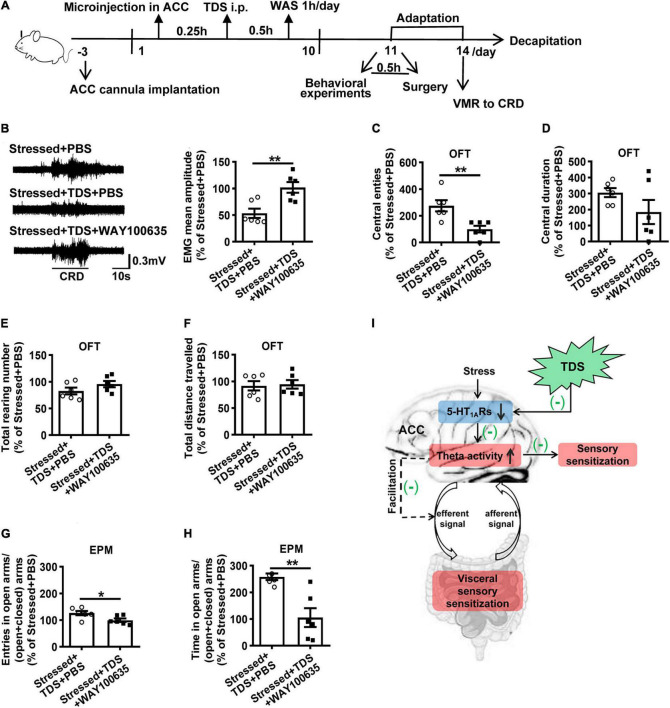
The effects of TDS in stress-induced visceral hypersensitivity were mediated by 5-HT_1A_Rs. **(A)** Experimental design. **(B)** The representative EMG of visceromotor response to 60 mmHg CRD (left) and the mean amplitude of EMG (right). Analysis of the central entries **(C)**, central duration **(D)**, the total raring number **(E)**, the total distance travelled **(F)** in the OFT. Analysis of the percentage of open arms entries **(G)** and the percentage of time spent into open arms **(H)** in the EPM. **(I)** Schematic summary of mechanisms underlies tandospirone alleviating visceral hypersensitivity induced by stress. Stress-induced 5-HT_1A_R downregulation leads to ACC activity increase, which is expressed as theta oscillation enhancement responding to visceral extension. Increased theta oscillations may result from over-responsive ACC to visceral afferent stimuli, thus manifesting central pain sensitization. Besides, theta oscillations increase may trigger descending pathway facilitation, thus increasing visceral sensitivity in the colon and contributing to visceral hypersensitivity. TDS activates 5-HT_1A_Rs and inhibits theta oscillation enhancement in ACC to ameliorate visceral hypersensitivity may *via* the above two pathways. (*n* = 6 for Stressed + PBS, Stressed + TDS + PBS and Stressed + TDS + WAY100635 respectively). *p < 0.05, **p < 0.01. Data are presented as means ± SEM.

As a partial 5-HT_1A_Rs agonist, TDS also has a low affinity toward other receptors, such as 5-HT_2_Rs, dopamine D2, and D4 receptors (Hamik et al., 1990; Nishimura et al., 2001). To identify whether TDS relieved visceral hypersensitivity by activating 5-HT_1A_Rs of ACC in stressed rats, we further examined VMR to CRD and anxiety-like behavior in stressed rats with TDS intraperitoneal injection and 5-HT_1A_R antagonist (WAY100635) or PBS microinjection in ACC. The data were shown as percentages relative to the value of stressed + PBS rats. Compared with the stressed + TDS + PBS rats, an additional application of WAY100635 targeting ACC abolished the effects of TDS in VMR amplitude (Stressed + TDS + PBS: 53.5 ± 8.8% vs. Stressed + TDS + WAY100635: 101.9 ± 10.1%, *p* < 0.01; Figure 5B). In behavioral tests, WAY100635 application reduced the effects of TDS in central entries (Stressed + TDS + PBS: 275.0 ± 41.8% vs. Stressed + TDS + WAY100635: 100.0 ± 25%, *p* < 0.01; Figure 5C) in the OFT, the percentage of number of open arms entries (Stressed + TDS + PBS: 126.5 ± 8.2% vs. Stressed + TDS + WAY100635: 99.7 ± 6.7%, *p* < 0.05; Figure 5G) and the percentage of time spent in open arms (Stressed + TDS + PBS: 258.5 ± 12.3% vs. Stressed + TDS + WAY100635: 105.6 ± 35.4%, *p* < 0.01; Figure 5H) in the EPM. These results suggest that TDS ameliorates visceral hypersensitivity and anxiety-like behavior of stressed rats by activating 5-HT_1A_Rs in ACC.

## Discussion

In this study, we found that downregulated 5-HT_1A_Rs enhanced ACC theta oscillations in visceral hypersensitivity induced by stress. TDS relieved visceral hypersensitivity and anxiety-like behavior induced by stress, which may relate to its activation of 5-HT_1A_Rs in ACC. 5-HT_1A_Rs activation inhibited the enhancement of theta oscillations in ACC, thus contributing to the amelioration of visceral hypersensitivity and anxiety induced by stress.

Serotonin is an important neurotransmitter that is involved in the peristaltic reflex of the gastrointestinal tract and pathological states such as pain disorders, anxiety, and depression (Primmer et al., 2010). Its role in IBS is widely acknowledged. Medicines targeting certain 5-HT receptors have been approved for IBS. For example, alosetron, an antagonist of 5-HT_3_ receptors, is effective for patients with IBS-diarrhea. However, due to serious side effects, such as constipation and ischemic colitis, it is only used under restrictive guidelines (Delvaux et al., 1998; Mayer et al., 2002). Moreover, tegaserod, a selective partial agonist of the 5-HT_4_ receptors, was used to improve symptoms in patients with IBS-constipation, but it has been withdrawn due to potential cardiovascular side effects (Coffin et al., 2003; Hoffman et al., 2012). Other agents such as 5-HT_4_ receptor agonists (prucalopride, ATI-7505, TD-5108) and 5-HT_3_ receptor antagonists (ramosetron) are being tested for the treatment of patients with IBS (Qin et al., 2014). As a 5-HT_1A_Rs partial agonist, TDS acts on the central 5-HT system to exert its anti-anxiety effect (Evans et al., 1994; Kawana et al., 2010). Clinical studies have demonstrated that the combinative application of TDS relieves gastrointestinal symptoms and anxiety in patients with IBS (Lan et al., 2014). Consistently, in this study, we found that TDS ameliorated the visceral hypersensitivity and anxiety-like behavior induced by stress in rats.

5-HT_1A_Rs, as the inhibitory serotonergic receptors, are critical in stress-related visceral hypersensitivity (Akimova et al., 2009). Furthermore, it has been proposed that 5-HT_1A_R-mediated stress moderation plays an important role in the function of 5-HT in the facilitation of adaptive responses to adversity in the brain (Carhart-Harris and Nutt, 2017). A PET study in humans revealed that chronically stressed subjects displayed a reduction of 5-HT_1A_Rs binding in the ACC, IC, and hippocampus when compared to non-stressed subjects (Jovanovic et al., 2011). Moreover, research in marmoset monkeys found that parental separations in infancy affected 5-HT_1A_Rs binding in ACC (Law et al., 2009). In this study, we also found 5-HT_1A_R downregulation in ACC of stressed rats with visceral hypersensitivity and anxiety behavior. However, the increase of 5-HT concentration in stressed rats could be an adaptive response to compensate for the impaired function of 5-HT_1A_Rs. One hypothesis for the 5-HT increase is that besides suppressing 5-HT_1A_R expression, anxiety stress may also inhibit 5-HT re-uptake in ACC, resulting in the 5-HT concentration increase in ACC. Moreover, stress may also downregulate the auto-5-HT_1A_Rs in the dorsal raphe nucleus, thus facilitating 5-HT release from their terminals in ACC to increase 5-HT concentration. Additionally, other mechanisms’ involvement could not be ruled out.

In the brain, regular and synchronized neuronal activity generates oscillations, which are a hallmark of neuronal network function. Theta oscillations, as the prominent feature of neural activity, are proposed to be a potential neural mechanism underlying visceral pain (Stern et al., 2006; Tai et al., 2006; Wang et al., 2015). An electroencephalogram study in neurogenic pain patients revealed theta and beta oscillations were over-activated in the pain matrix including ACC (Stern et al., 2006). Besides, an animal study showed that repeated noxious visceral stimulation in rats enhanced endogenous theta rhythms in the ACC. At the same time, cross-correlation analysis revealed augmented synchronization of theta oscillations in the thalamus and ACC (Wang et al., 2015). In this study, we found enhanced theta band LFPs in ACC of stress-induced visceral hypersensitivity rats, too. Furthermore, the power of theta oscillations in ACC was found to be positively correlated with the level of visceral sensitivity, leading to the suggestion that enhanced theta oscillations in ACC may underlie visceral hypersensitivity.

It has been shown that 5-HT_1A_Rs are involved in the regulation of theta oscillations. For example, a study in awake cats found that preferential activation of presynaptic 5-HT_1A_Rs facilitated the appearance of theta activity in the hippocampus (Marrosu et al., 1996). Horváth et al. (2015) reported that buspirone, another agonist of 5-HT_1A_ receptors, reduced anxiety-like behavior in rats while increasing hippocampal theta oscillatory frequency. In addition, an *in vitro* study showed that perfusion with the 5-HT_1A_Rs antagonist enhanced the theta band oscillatory activity in hippocampal slices (Kazmierska and Konopacki, 2015). The inconsistent results could be attributed to differences in animal species, brain regions, or affinity to receptors of different drugs. In this study, we demonstrated the reduced 5-HT_1A_Rs expression with enhanced theta oscillations in ACC of stressed rats. More importantly, activation of 5-HT_1A_Rs by its agonist, 8-OH-DPAT, to compensate for the effect of 5-HT_1A_Rs, reduced the enhancement of theta oscillations in ACC induced by stress. As a partial 5-HT_1A_Rs agonist, TDS also mimicked the effect of 8-OH-DPAT to activate 5-HT_1A_Rs and suppressed the enhancement of stress-induced theta oscillations in ACC. Furthermore, the fact that selective 5-HT_1A_R antagonist targeting ACC blocked effects of TDS in visceral hypersensitivity and anxiety-like behavior induced by stress demonstrated that effects of TDS in stressed rats were mediated by activating 5-HT_1A_Rs in ACC but not in other receptors.

The question arises of how enhanced ACC theta band activation induces visceral pain sensitization. ACC, as a critical central nervous system (CNS) pain center, has wide connections with the limbic system, autonomic effector areas, and centers of arousal and pain modulation (Mertz et al., 2000). It receives nociceptive sensory inputs from the thalamus, amygdala, and other pain-related areas of the cortex. Meanwhile, ACC neurons project to the hypothalamus, periaqueductal gray, spinal cord, prefrontal cortex, and other cortical and subcortical structures to mediate sensory modulation, anxiety, fear, and memory (Bliss et al., 2016). As a result, ACC may mediate the “emotional” content of nociceptive sensory input. As a G-protein-coupling receptor in the 5-HT system, 5-HT_1A_R is activated by 5-HT and causes neuronal hyperpolarization and excitability decrease by activating an inwardly rectifying K^+^ channel *via* G protein-mediated pathway (Bayliss et al., 1997). Therefore, 5-HT_1A_R activation in the ACC inhibits neuronal activity in this region, whereas 5-HT_1A_R inhibition promotes ACC activity. In stressed rats, 5-HT_1A_R expression and its effects were downregulated in the ACC. Downregulated 5-HT_1A_Rs mediated neural activity increases responding to visceral extension in ACC of stressed rats, expressed as theta oscillation enhancement, which may be resulted from over-response of ACC to visceral afferent stimuli due to increased excitability of ACC neurons. On the other hand, ACC sensitization mediated by downregulated 5-HT_1A_Rs may trigger descending pathway facilitation, thus increasing visceral sensitivity in the colon and contributing to visceral hypersensitivity in stressed rats. Besides the effect of tandospirone in CNS, there are other studies that reported that tandospirone may regulate brain-gut response and have an anti-spasmodic effect on the colon by binding 5-HT_*lA*_Rs, thereby producing an improvement in abdominal pain and diarrhea in patients with IBS (Lan et al., 2014). Thus, the effect of tandospirone on the enteric nervous system could not be ruled out and deserves further study in the future.

This study was conducted only on male rats to avoid the potential impacts of menstrual cycles on visceral pain. However, the experimental design with single-sex limits the generalization of this observation to female subjects. Given that IBS is a female predominant syndrome and female sex hormones are related to the severity of IBS symptoms (Heitkemper and Chang, 2009; Lenhart and Naliboff, 2020), the sex difference in TDS effects is important and merits further investigation. In this study, as we found that the level of anxiety of sham-WAS (same treatment as WAS but in the tank without water) rats varied between individuals due to different stressful responses of rats to the elevated vertical platform, we used normal control instead of sham-WAS as the control group to avoid the potential impacts this variability on the results, which is similar to other studies (Contuk et al., 2005; McGonagle et al., 2012; Hassan et al., 2014; Wang et al., 2017; Yu et al., 2018; West et al., 2021; Gurel and Sogut, 2022). Besides, because the chronic TDS treatment and stress were carried out concurrently, there was a period of time without TDS administration between stress and visceral sensitivity measurement. This drug-free period may have reduced the effect of TDS, but TDS still had significant effects. Nevertheless, despite the above limitations, our results provide evidence showing that downregulation of 5-HT_1A_Rs mediated enhanced theta oscillations in ACC, which is involved in the formation of stress-induced visceral hypersensitivity. TDS ameliorates visceral hypersensitivity and anxiety-like behavior in stress-induced visceral hypersensitivity rats by activating 5-HT_1A_Rs to suppress the enhancement of theta oscillations in ACC induced by stress. Our results provide evidence for a novel mechanism of regulating the 5-HT_1A_R–theta-oscillation pathway, whereby TDS relieves anxiety and visceral pain. Given that anxiety is a common psychiatric comorbidity of IBS and involves the formation and aggravation of IBS symptoms, this study may have important implications for combinative use of tandospirone with other medicines for patients with IBS with anxiety to enhance therapeutic outcomes. Moreover, as visceral pain potentially shares similar central mechanisms, this effect, as well as its mechanisms, may be of general importance for other visceral pain, such as pelvic pain or vulvodynia.

## Data availability statement

The original contributions presented in this study are included in the article/supplementary material, further inquiries can be directed to the corresponding authors.

## Ethics statement

The animal study was reviewed and approved by the Animal Care and Use Committee of Tongji University.

## Author contributions

YH and S-CX designed and directed the research. T-TZ and Z-YD performed a majority of the experiments and wrote the manuscript. L-SY, YZ, and H-QZ performed the experiments. H-HS, J-WW, and YC planned the experiments and provided technical assistance. All authors contributed to the article and approved the submitted version.
